# Supplementary material for: Identification of Peanut AhMYB44 Transcription Factors and Their Multiple Roles in Drought Stress Responses
Source: Plants (Basel). 2022 Dec 14;11(24):3522. doi: 10.3390/plants11243522 (PMC9788490; doi:10.3390/plants11243522)
Supplement: Supplementary file 1 [file plants-11-03522-s001.zip › Figures.pdf]

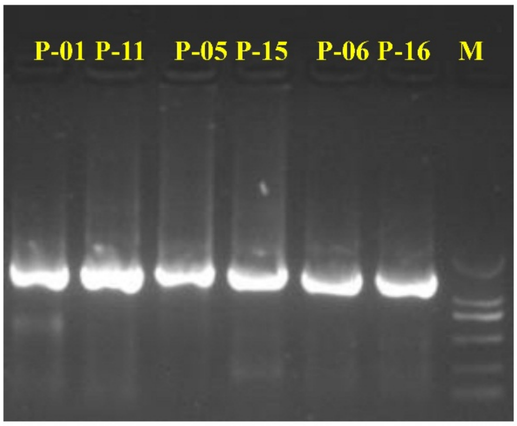

Figure S1. PCR products of the promoter of *AhMYB44s*.

P-01/11/05/15/06/16: promoter of *AhMYB44-01/11/05/15/06/16*, M: DL2000. Band sizes were about 1500bp.

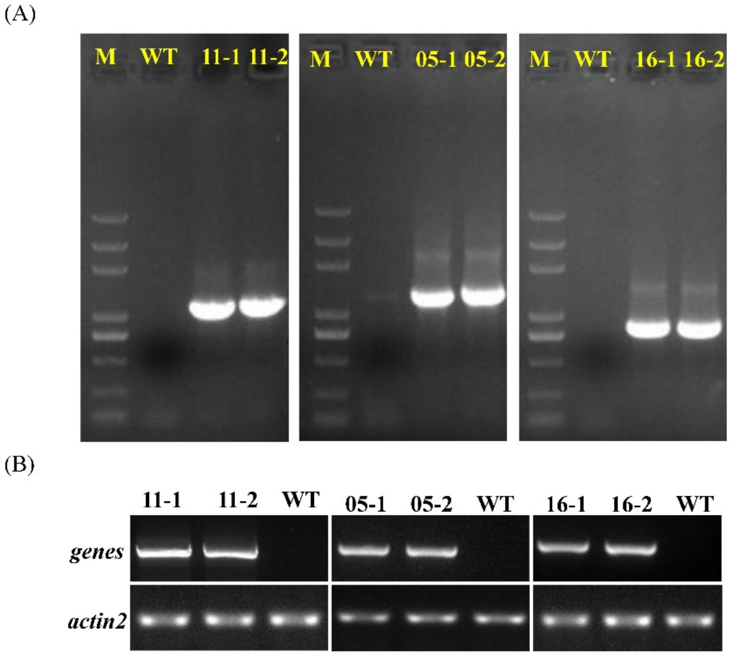

Figure S2. Analysis of the transgenic plants by PCR.

(A). Selection of trans-formants by PCR. M: AL5000; WT: wild-type *Arabidopsis*; 11-1/2 (11-OE1/2): the two lines of *AhMYB44-11* transgenic *Arabidopsis*. Band sizes were about 950bp. 05-1/2 (05-OE1/2): the two lines of *AhMYB44-05* transgenic *Arabidopsis*. Band sizes were about 1100 bp.16-1/2 (16-OE1/2): the two lines of *AhMYB44-16* transgenic *Arabidopsis*. Band sizes were about 760 bp. (B). Transcript levels of the transgenic *Arabidopsis* and WT plants assayed by semi-quantitative reverse transcription PCR. *AtACTIN2* was used as an internal control.

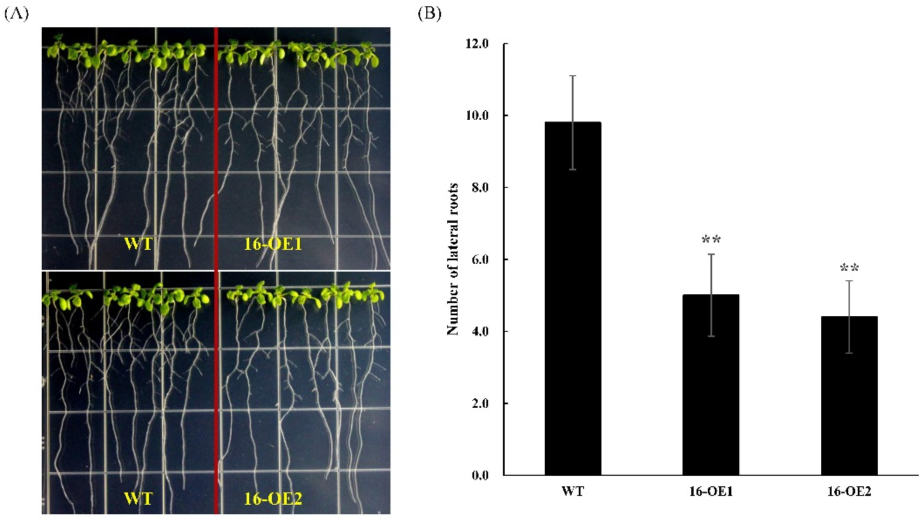

Figure S3. Difference of lateral roots between WT and 16-OEs under normal condition.

(A). Phenotype of WT and 16-OEs grown vertically on 1/2 MS medium for about 10 days.

(B). Number of lateral roots (≥1mm) of WT and 16-OEs seedlings. ** $p < 0.01$.
