# Supplementary material for: Identification of Peanut AhMYB44 Transcription Factors and Their Multiple Roles in Drought Stress Responses
Source: Plants (Basel). 2022 Dec 14;11(24):3522. doi: 10.3390/plants11243522 (PMC9788490; doi:10.3390/plants11243522)
Supplement: Supplementary file 1 [file plants-11-03522-s001.zip › Table S2.pdf]

Table S2 Primers used in this study

Name	Forward primer sequence	Reverse primer sequence	Experiments
AhMYB44-01	5’-CCTTACCTACTTCGCTTCT-3’	5’-CATTTCCTACTAATAACACCC-3’	gene isolation
AhMYB44-11	5'-CTTTATGGTCTTGTCTTCGT-3'	5'-CTTTTCTTATCATTTCTTGC-3'	gene isolation
AhMYB44-05	5’-CCCCACCCTTTATTTTAT-3’	5’-AACAGGGGATAAACACGA-3’	gene isolation
AhMYB44-15	5'-ACCACCAACACAACTAACAC-3'	5'-AACAGGGGATAAACACGAAG-3'	gene isolation
AhMYB44-06	5'-TTATCTTTCCGTTTTCCCCA-3'	5'-TTTTTACCTCTCTAATCCGT-3'	gene isolation
AhMYB44-16	5’-GTGCTCTGTTTCGCTTTT-3’	5’-AAACTGTTCCTGCTCCAA-3’	gene isolation
Pro-MYB44-01	5’-TATTGCCTTTGTTTCATACAACGAA-3’	5’-ACTGCTTAGCTTCCTTTGTATTG	promoter isolation
Pro-MYB44-11	5’-GTTCTTGTAACAACTTGAGGC-3’	5’-AGTGCTTAGTTTCTTTTGTTTTG-3’	promoter isolation
Pro-MYB44-05	5’-GTACGAGTAACAGAAATAGATACGG-3’	5’-CTCTCTCTTATCTTTTTTTCCTCC-3’	promoter isolation
Pro-MYB44-15	5’-TGGGTAGTATATGTACGATGTACGA-3’	5’-CTCTCTCTTATCTTTTTTTCCTCCT-3’	promoter isolation
Pro-MYB44-06	5’-TCTGAATTAAACTCTGCCATTAGAC-3’	5’-TGCAATAAGAATCAAATCAAATTCC-3’	promoter isolation
Pro-MYB44-16	5’-ATCAATACTAAAGCAAAACCACAAT-3’	5’-TGCAATAAGAATAAAATCAAATTCC-3’	promoter isolation
Q-MYB44-01/11	5’-ATTGCCATCGCCACAACATC-3’	5’-CACCAGCAGCCTGAAAACACAT-3’	qRT-PCR
Q-MYB44-05/15	5’-CCGTTGACTGCCATACCTCTG-3’	5’-CCGCTCCATTATTCTGCTTCG-3’	qRT-PCR
Q-MYB44-06/16	5’-GTGCCGCTCTTCTGCTTCTGA-3’	5’-CGGGCTGCTAGGCGTGGTAA-3’	qRT-PCR
Q-AhACTIN11	5’-TTGGAATGGGTCAGAAGGAT-3’	5’-AGTGGTGCCTCAGTAAGAAGC-3’	qRT-PCR
Q-AtACTIN2	5’-GGTAACATTGTGCTCAGTGGTGG-3’	5’-AACGACCTTAATCTTCATGCTGC-3’	qRT-PCR
Q-AtPP2CA	5’-TCTGAGAATCATCATTTCTACGGTGT-3’	5’-CTCGTTGGCTAACTTCTTTATCCATT-3’	qRT-PCR
Q-AtABI1	5’-AGAAACCGATGCTCTGCGATGGTGATAC-3’	5’-GTTAGCGACGAAGATGTGAGACGGG-3’	qRT-PCR
Q-AtABA1	5’-GATTCTGGAGATAAGGTTACTGTGG-3’	5’-CCGTGTAACAAGTGTAGCCTGAAT-3’	qRT-PCR
Q-AtABA2	5’-GCTTGTCTGTGTCAATAGTGAGGCA-3’	5’-GATCAGAGGAAAAAGTTGGAGAAAG-3’	qRT-PCR
Q-AtP5CS1	5’-TCTTATGGCTTACTATGAGACTATGTTTGAC-3’	5’-TATGGGGCTCTTCGGGTGCTAATAG-3’	qRT-PCR
Q-AtRD29A	5’-GTCTGCCGTGACGACGAAGTTAC-3’	5’-TCCTTCTTCTCTTCTTCTCCTCCAA-3’	qRT-PCR
Q-AtCBF1	5’-TGTCTCAACTTCGCTGACTCGGC-3’	5’-ACCTTCGCTCTGTTCCGGTGTATAA-3’	qRT-PCR
Q-AtCOR15A	5’-GGTAAAGCAGGAGAGGCTAAGGATG-3’	5’-AAGAATGTGACGGTGACTGTGGATA-3’	qRT-PCR
BD-MYB44-05	5’-TGGCCATGGAGGCCGAATTCATGAATACCGCTACTAGT-3’(EcoR1)	5’-CGCTGCAGGTCGACGGATCCTTACGAATCGATCCT-3’(BamH1)	Transcriptional activity
BD-MYB44-11	5’-TGGCCATGGAGGCCGAATTCATGGCCATGTCTAGA-3’(EcoR1)	5’-CGCTGCAGGTCGACGGATCCTCACGAGTTGACTTTG-3’(BamH1)	Transcriptional activity

BD-MYB44-16	5'-TGGCCATGGAGGCCGAATTCATGGATCGAGTTAAG-3'(EcoR1)	5'-CGCTGCAGGTCGACGGATCCTTACCTCTCTATTCC-3'(BamH1)	Transcriptional activity
SL-MYB44-05	5'-GCTCTAGAATGAATACCGCTACTAGTA-3'(XbaI)	5'-GGGGTACCCGAATCGATCCTGCTAATC-3' (KpnI)	Subcellular localization
SL-MYB44-11	5'-GGACTAGTATGGCCATGTCTAGAAGAG-3' (SpeI)	5'-GGGGTACCCGAGTTGACTTTGCTAATC-3' (KpnI)	Subcellular localization
SL-MYB44-16	5'-GCTCTAGAATGGATCGAGTTAAGGGC-3'(XbaI)	5'-GGGGTACCCCTCTCTATTCCGTTTCTA -3'(KpnI)	Subcellular localization
TS-MYB44-05	5'-CCCAAGCTT ATGAATACCGCTACTAGTA-3'(HindIII)	5'-GGGGTACC TTACGAATCGATCCTGC-3'(KpnI)	Transformation
TS-MYB44-11	5'-CCCAAGCTTATGGCCATGTCTAGAAG-3'(HindIII)	5'-GACTAGTTCACGAGTTGACTTTG-3'(SpeI)	Transformation
TS-MYB44-16	5'-CCCAAGCTT ATGGATCGAGTTAAGG-3'(HindIII)	5'-GACTAGTTTACCTCTCTATTCC-3'(SpeI)	Transformation
